# Extracting DNA words based on the sequence features: non-uniform distribution and integrity

**DOI:** 10.1186/s12976-016-0028-3

**Published:** 2016-01-25

**Authors:** Zhi Li, Hongyan Cao, Yuehua Cui, Yanbo Zhang

**Affiliations:** Department of Health Statistics, School of Public Health, Shanxi Medical University, Taiyuan, 030001 China; Department of Statistics and Probability, Michigan State University, East Lansing, MI 48824 USA

**Keywords:** DNA words, DNA vocabulary, Integrity of word

## Abstract

**Background:**

DNA sequence can be viewed as an unknown language with words as its functional units. Given that most sequence alignment algorithms such as the motif discovery algorithms depend on the quality of background information about sequences, it is necessary to develop an ab initio algorithm for extracting the “words” based only on the DNA sequences.

**Methods:**

We considered that non-uniform distribution and integrity were two important features of a word, based on which we developed an ab initio algorithm to extract “DNA words” that have potential functional meaning. A Kolmogorov-Smirnov test was used for consistency test of uniform distribution of DNA sequences, and the integrity was judged by the sequence and position alignment. Two random base sequences were adopted as negative control, and an English book was used as positive control to verify our algorithm. We applied our algorithm to the genomes of *Saccharomyces cerevisiae* and 10 strains of *Escherichia coli* to show the utility of the methods.

**Results:**

The results provide strong evidences that the algorithm is a promising tool for ab initio building a DNA dictionary.

**Conclusions:**

Our method provides a fast way for large scale screening of important DNA elements and offers potential insights into the understanding of a genome.

**Electronic supplementary material:**

The online version of this article (doi:10.1186/s12976-016-0028-3) contains supplementary material, which is available to authorized users.

## Background

Like a sealed book, the genome contains all information in its sequences and extracting the words of the language is a key step to decrypt the secret of life. A lot of sequence alignment algorithms, such as motif discovery algorithms [1, 2], were developed for this purpose. However these algorithms are limited in two ways: 1) their performances depend on the quality of available background information about the sequences, that is the extent of knowledge about biological function [1]; and 2) they can not analyze genomic regions with unknown functions. Therefore, it is necessary to develop an ab initio algorithm for extracting meaningful DNA words based only on DNA sequence itself.

Some ab initio methods have been developed in the literature, such as k-mer [3], relative entropy [4], and information content [5–8]. In these methods, the frequency information of a word in a DNA sequence was used widely, but the position information was not paid enough attention. However, the position information, i.e., the distribution of a word in a DNA sequence, is very important to understand the function of the elements. A uniform distribution can be assumed when no information can be obtained from the distribution of a sequence [9]. Therefore, the basic hypothesis in this study is that the information of a functional DNA element deviates from a uniform distribution. Based on this hypothesis, identifying patterns of DNA sequences that deviate from uniform distribution can provide a fast means for word detection and shed novel light on the function of a genome. In addition, the integrity is also an important feature of a word [10]. Integrity means that a word should be a complete unit which has a clear boundary. For example, the statistical characteristics (e.g., distribution or frequency) of “biology” and “iology” are almost the same in an English text. But the former is a word because it is a complete unit and has particular meaning. Currently, hardly any algorithm can distinguish them based purely on DNA sequences. Based on the two features, we can define a word as a complete symbol sequence not following the uniform distribution within a certain scope.

Carpena et al. [11] have shown the importance of the distribution in the identification of words. In their work, a clustering coefficient was used to denote the distribution of words. In addition, the semantic meanings of the words were used to explore the integrity. However, the semantic meanings are difficult to be applied for genome sequences due to the lack of a dictionary to define the genome content. Hackenberg et al. [12] also applied a clustering coefficient to denote the distribution of a base sequence within a one-dimensional DNA sequence context. Their results showed that the clustering of a DNA word was significantly associated with functional elements. However, no methods were provided to check the integrity, which may lead to false positives.

In this study, we developed an algorithm to extract meaningful DNA words based on these two features: non-uniformity and integrity. A Kolmogorov-Smirnov (KS) test was used for consistency test of uniform distribution, and the integrity of a word was checked by the sequence and position alignment among the symbol sequences. To verify the algorithm, both negative and positive controls were considered. In principle, a random sequence following a uniform distribution should not carry any information, and nothing can be found according to this algorithm in principle. Thus, two random sequences were used to check the false positives. We also used an English book as a positive control to check if the algorithm can identify meaningful words without specifying any structures. Finally, we applied our method to the genomes of *Saccharomyces cerevisiae* (Scere) and 10 strains of *Escherichia coli* (Ecoli). Results show that DNA words extracted from a DNA strand can carry specific information to reveal biological functions. The identified DNA words can be incorporated into a DNA vocabulary, based on which and in coupling with gene function information derived from Gene Ontology (GO) database, we can explore the relationships between these DNA words and gene functions via fast computational tools.

## Methods

### Statistical tests

#### Consistency test of uniform distribution

In this study, the KS test was used for consistency test of uniform distribution in DNA sequences. As shown in equation (1), *F*(*x*) is defined as the cumulative distribution function of a uniform distribution, *F*_*n*_(*x*) is the empirical distribution function of the sample, and *D*_*n*_ quantifies a distance between them.

1 $$ {D}_n= max\kern0.5em \left|{F}_n\kern0.5em (x)-F(x)\right| $$

For example, in the chromosome NC_009786.1 (79237 bp) shown in Section 4, the base T appears 20384 times, and its positions on this chromosome can be recorded as follows. (For avoiding unreasonable segmentation, the full length of every chromosome was analyzed.)

**Table:** 

NC_009786.1:	T	T	C	A	G	A	T	T	A	A	…
Positions of T:	1	2					7	8			…

Based on these positions, a *P* value can be inferred by the KS test. The number of the positions, 20384, is the sample size. Similarly, the positions of an arbitrary repeated sequence in a genome can be recorded, and whether or not it is evenly distributed in the genome can also be tested.

According to the basic hypothesis, the closer the distribution of a sample is to a uniform distribution, the less the information it carries and the smaller the *D*_*n*_ value is. Considering the practical significance of the information, it was necessary to set a threshold for *D*_*n*_. Our preliminary study [13] had shown that considering the restrictions of the sample size and the sampling error, the minimum *D*_*n*_ value can be set as 0.1, and the sample size should be larger than 100.

### Judgement of integrity

Assume that the sets *s*_*1*_, *s*_*2*_, …, *s*_*n*_ were the positions of the respective words *w*_*1*_, *w*_*2*_, …, *w*_*n*_ in the same chromosome. Here the positions of each word include all the physical locations occupied by the word. Then, the integrity can be described as equation (2).

2 $$ {s}_1\cap {s}_2\cap \cdots \cap {s}_n=\varnothing $$

The integrity means that the boundary of a word can be identified. For example, “bioinformation” is an English word, and its subsequence “ioinformatio” should not be identified because its boundary is wrong. But its subsequence “information” is a different English word and should be identified. Then how to filter the results like “ioinformatio”. In an English text, “ioinformatio” should appear at the positions of “bioinformation”, therefore the positions of “ioinformatio” would be filtered after the positions of “bioinformation” are deleted. But “information” is different. On the one hand, it is a subsequence of “bioinformation”, therefore it can appear at the positions of “bioinformation”. On the other hand, it is also an English word, therefore it can appear at the positions not belonging to “bioinformation”, too. In this algorithm, the total of two classes of positions are named as raw positions, and the second class are named as net positions. According to equation (2), the net positions of “information” should be tested whether or not they are evenly distributed.

## The algorithm for extracting words

**Fig. 1:**
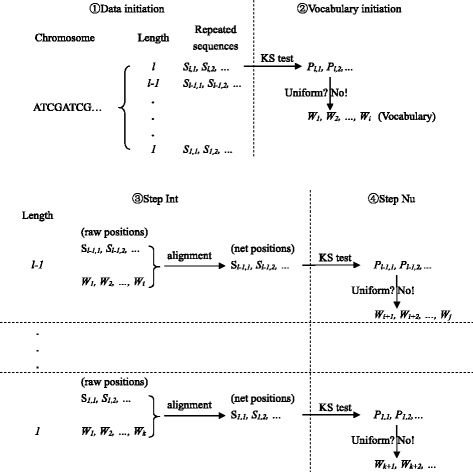
Illustration of extracting words. “*S*_*l*,*1*_” represented the positions of the first repeated sequence of length *l*, and “*P*_*l*,*1*_” represented its *P* value for KS test, and so on. “*W*_*1*_” represented the positions of the first recognized word, and so on

By combining the two criteria stated above, namely, non-uniformity and integrity, we developed an algorithm named Nu-Int (Non-uniform & Integrity) to extract meaningful DNA words from a DNA strand. As shown in Fig. 1, this algorithm includes five steps summarized as follows.

1. Data initialization: All symbol sequences whose numbers of repetitions > 100 were extracted and classified by their lengths.
2. Vocabulary initialization: In the beginning, the vocabulary of the DNA strand is null. Therefore, we do not need to check equation (2). The symbol sequences in the class with the longest length were tested separately by the KS test. The type I error *α* was adjusted according to the formula, *α* = 1–0.95^(1/*n*), where *n* is the number of symbol sequences in this class. When *P* < *α* and *D*_*n*_ > 0.1, it can be considered that the symbol sequence did not follow the uniform distribution, and can be added in the vocabulary of the DNA strand.
3. Step Int: The symbol sequences in the next class were compared with the recognized words in the vocabulary. If a symbol sequence was the subsequence of a recognized word, the positions of this word should be deleted from the raw positions of this symbol sequence.
4. Step Nu: The net positions of this symbol sequence were tested by the KS test. The symbol sequences not meeting the requirements (i.e., numbers of repetitions > 100, *P* < *α* and *D*_*n*_ > 0.1) would be eliminated.
5. Repeating step (3)-(4). The symbol sequences in the rest classes were analyzed in descending order of word lengths.

In this study, the sequence and position alignment among the symbol sequences was executed by Perl language, and the KS test was executed by R language [14].

## Algorithm verification with control

### Negative control

In this study, two pseudo chromosomes (rand1 and rand2) were used to evaluated the denoising ability of this algorithm. They were made up of the random arrangements of bases with equal probability, and their length were 4,000 bp and 6,400,000 bp, respectively.

### Positive control

Unlike natural language, there is not a reference vocabulary in the genome. Therefore we can not verify directly the result extracted from the genome. However, the comma-less texts from natural language is a good analogy [12]. It was relatively easy to identify whether or not the result extracted from the English text was a word. In this study, an English book, the *Holy Bible* (King James), was adopted as a positive control. In this text, only 26 letters of the English alphabet were retained, and all uppercase letters were converted to lowercase. The number of remaining characters in this book was 3,317,198.

## Algorithm verification with genomic comparison

Due to limited knowledge, we could not distinguish the meanings of the extracted DNA words directly. But the DNA words from different genomes can be used to show the difference among the genomes. In this study, we downloaded ten strains of Ecoli genomes from the NCBI website [GenBank: NC_000913.2, NC_007779.1, NC_010473.1, NC_009801.1, NC_009786.1, NC_009787.1, NC_009788.1, NC_009789.1, NC_009790.1, NC_009791.1, NC_011353.1, NC_011350.1, NC_011351.1, NC_011415.1, NC_011407.1, NC_011408.1, NC_011411.1, NC_011413.1, NC_011416.1, NC_011419.1, NC_011741.1, NC_012967.1, NC_012971.2, NC_016902.1, NC_016903.1, NC_016904.1] and Scere genome [GenBank: NC_001133.9, NC_001134.8, NC_001135.5, NC_001136.10, NC_001137.3, NC_001138.5, NC_001139.9, NC_001140.6, NC_001141.2, NC_001142.9, NC_001143.9, NC_001144.5, NC_001145.3, NC_001146.8, NC_001147.6, NC_001148.4, NC_001224.1]. The download links were given in Additional file 1. The sequences of the chromosomes were collected from the .fna files. Each chromosome was numbered, and the numbers were given in Additional file 2. The lengths of these chromosomes were listed in Table 1 and were sorted in ascending order.

**Table 1:** List of chromosomes and their lengths

Chromosome	Length(bp)	Chromosome	Length(bp)	Chromosome	Length(bp)
Ecoli06_pla3.fna^a^	4082	Ecoli06_pla6.fna	100021	Scere07.fna	1090940
Ecoli04_pla6.fna	5033	Ecoli10_pla2.fna	103795	Scere15.fna	1091291
Ecoli10_pla1.fna	5360	Scere01.fna	230218	Scere04.fna	1531933
Ecoli06_pla2.fna	5366	Scere06.fna	270161	Ecoli09.fna	4558953
Ecoli04_pla4.fna	6199	Scere03.fna	316620	Ecoli08.fna	4629812
Ecoli06_pla1.fna	6929	Scere09.fna	439888	Ecoli01.fna	4639675
Ecoli04_pla2.fna	34367	Scere08.fna	562643	Ecoli02.fna	4646332
Ecoli05_pla2.fna	37452	Scere05.fna	576874	Ecoli03.fna	4686137
Ecoli06_pla5.fna	60555	Scere11.fna	666816	Ecoli07.fna	4700560
Ecoli04_pla3.fna	70609	Scere10.fna	745751	Ecoli06.fna	4887515
Ecoli04_pla5.fna	74224	Scere14.fna	784333	Ecoli10.fna	4920168
Ecoli04_pla1.fna	79237	Scere02.fna	813184	Ecoli04.fna	4979619
Scere_mit.fna	85779	Scere13.fna	924431	Ecoli05.fna	5572075
Ecoli06_pla4.fna	91158	Scere16.fna	948066		
Ecoli05_pla1.fna	94644	Scere12.fna	1078177		

^a^ “pla” represented plasmid

All 43 chromosomes were analyzed, and 86 vocabularies for all DNA strand were established. The DNA words extracted from those DNA strands of Ecoli can compose an Ecoli vocabulary. Similarly, the Scere vocabulary can also be built. The difference between the chromosomes can be illustrated by comparing these vocabularies.

## Associations between DNA words and gene functions

All DNA words extracted from these genomes were incorporated into a DNA vocabulary. Gene sequences were extracted from .fna files according to the corresponding sites provided by .gbk files, and the word frequency of each DNA word in a gene can be counted. Gene annotations from the GO were adopted for this study, and the categories of gene functions can be collected. The download links of the Ontology files and the gene annotations of Ecoli and Scere were given in Additional file 1. Based on these data, a logistic regression was used to investigate the relationships between the categories of gene functions and the DNA words.

### Grading GO terms according to Ontology file

For enriching the genes annotated by similar functions, it is necessary to grade GO terms. The relationship among GO terms is a directed acyclic graph. All GO terms are divided into three categories (molecular function, biological process, and cellular component), and each category has a root node. The terms (IDs of functions) in the molecular function category were adopted in this study.

These terms were graded according to the GO hierarchy. The level–1 terms should be the direct child node of the root node and the level–2 terms were the direct child node of the level–1 terms, and so on. For enriching all annotated genes of a term, a gene annotated by its child terms should be enriched, therefore its all child term, including direct and indirect ones, should be recorded.

### Grouping genes according to gene annotations

To get gene annotations, the function entries were extracted from the annotation files, and the entries with evidence code IEA were deleted. The genes appeared in these entries were associated with the genes in .gbk files, and the genes with conflict identifications (37 genes from Ecoli, and 20 genes from Scere) were deleted.

Because the definitions of functions are not exclusive in Geng Ontology, a gene can be associated with several functions that belong to the same level in the directed acyclic graph. For each function, all annotated genes can be divided into three groups: exclusive, control and share. The exclusive group includes genes only annotated by the function; the control group includes genes not annotated by the function; the share group includes genes annotated simultaneously by other functions in the same level. The number of annotated genes of each group was recorded.

To facilitate the calculation, the functions whose numbers of annotated genes > 150 in the exclusive group and the control group were selected. Twenty nine GO terms satisfying the requirements were listed in Table 2.

**Table 2:** Groups of annotated genes by GO terms

GO terms	Exclusive	Share	Control	GO terms	Exclusive	Share	Control
Ecoli_l1_GO:0003824^a^	731	501	689	Scere_l2_GO:0016787	520	220	3344
Ecoli_l1_GO:0005215	224	46	1651	Scere_l2_GO:0022857	236	26	3822
Ecoli_l1_GO:0005488	367	562	992	Scere_l2_GO:0022892	236	42	3806
Ecoli_l2_GO:0016740	238	127	1556	Scere_l2_GO:0060090	329	18	3737
Ecoli_l2_GO:0016787	193	217	1511	Scere_l2_GO:0097159	731	407	2946
Ecoli_l2_GO:0022857	189	48	1684	Scere_l2_GO:1901363	729	400	2955
Ecoli_l2_GO:0022892	157	60	1704	Scere_l3_GO:0003676	399	369	3316
Scere_l1_GO:0003824	1513	294	2277	Scere_l3_GO:0016772	209	85	3790
Scere_l1_GO:0005198	274	83	3727	Scere_l3_GO:0016788	164	84	3836
Scere_l1_GO:0005215	303	41	3740	Scere_l3_GO:0016817	185	122	3777
Scere_l1_GO:0005488	1233	542	2309	Scere_l3_GO:0022891	164	35	3885
Scere_l2_GO:0003735	190	34	3860	Scere_l3_GO:0030533	298	0	3786
Scere_l2_GO:0005515	292	242	3550	Scere_l4_GO:0003723	278	166	3640
Scere_l2_GO:0016491	186	42	3856	Scere_l4_GO:0016301	154	33	3897
Scere_l2_GO:0016740	514	123	3447				

^a^ “l1” indicated that the term was a level–1 term, and so on

### Establishing the fitting models

The gene sequences were collected from the genomes based on their positions in .gbk files. One gene can appear on more than one chromosome, and its sequence might vary on different chromosomes. The word frequency of each DNA word was counted based on these gene sequences. Because the words of the DNA vocabulary varied in length, a short word can be the subsequence of a long word. In this case, the word frequency of this long word must be deleted from the word frequency of this short word. If the sequences of a gene varied, the average frequency was calculated, and the word frequency was divided by the number of sequences.

Based on the words frequency and genes in the exclusive group and the control group, the logistic regression was applied to investigate the relationships between the functions and the words. Two models, with or without interaction (equation 3 and 4), were adopted for each function. In the models, *Y*_*i*_ was the value of the *i*th gene associated with a function (1 for exclusive group, and 0 for control group), and *f*_*m*,*i*_ was the word frequency of the *m*th word in the *i*th gene.

3 $$ logit\left(E\left[{Y}_i\Big|{F}_i\right]\right)=\beta {F}_i={\beta}_0+{\beta}_1{f}_{1,i}+\cdots +{\beta}_m{f}_{m,i} $$

4 $$ logit\left(E\left[{Y}_i\Big|{F}_i\right]\right)=\beta {F}_i={\beta}_0+{\beta}_1{f}_{1,i}+\cdots +{\beta}_m{f}_{m,i}+{\beta}_{mn}{f}_{m,i}{f}_{n,i} $$

Because there were too many words in the vocabulary, a selection was necessary to simplify the analysis process. We considered two scenarios when performing the selection.

1. Without interaction
  Two selections were conducted. The first selection was done based on the word frequency. We defined *fe* as the word frequency in the exclusive group, *fc* as the word frequency in the control group, and *fd* = *fe* - *fc*. The words were selected if *fe* > 0.6 and *fd* > 0.1. The selected words were used to fit the logistic regression model. Due to the huge difference in the sample size between the two groups for most of the terms, bootstrapping was applied to estimate the statistics. In the second selection, *P* value was adopted as selection criteria. The words with *P* < 0.2 were chosen. The final model without interaction was built with these words.

(2) With first-order interaction
  The first selection was conducted as described above. If there were too many words selected from the first selection, there would be too many interaction terms in the model. Therefore, when the number of the words from the first selection was more than 50, the selection criteria of *fd* would raise until the number of selected words was less than 50. The second selection was executed the same as above. Subsequently, all first-order interaction terms of those chosen words were added in the model. Every interaction term was the product of two word frequencies. For the third selection, the terms with *P* < 0.2 were chosen. The final model with first-order interaction was established based on these chosen terms.

### Evaluating the fitting models by bootstrapping

Bootstrapping was used to estimate the predicted accuracy rate in the three groups (exclusion, control and share). We bootstrapped 100 samples with the size of each sample equal to the size of the original data set. Every gene can get a predicted probability *P* from the models. In the exclusive group, we thought that it was a correct prediction when *P* > 0.5, and the accuracy rate was defined as the sensitivity. In the control group, we thought that it was a correct prediction when *P* < 0.5, and the accuracy rate was defined as the specificity. Besides, the agreement rate of each function was also calculated. In the share group, it was right when *P* > 0.5. In this study, median absolute deviation (MAD) was adopted for robust measuring the variability of these indexes.

## Results

### Algorithm verification with control

For pseudo chromosomes (random sequences), no word was extracted by this method. The satisfied denoising ability was demonstrated by this result. It is possible that some DNA words can be generated by chance in these random base sequences. But their positions in these sequences should be random, and follow a uniform distribution. Therefore, they can not pass the selection criteria.

All 4523 words extracted from the *Holy Bible* were given in Additional file 3, and the top 20 longest English words were listed in Table 3. In this table, almost every word was made up of a few simple natural English words. These English words had clear meanings, and their meanings were related to the content of this book. Besides, the boundary of 65.7 % words can be identified accurately. For example, a compound word “thechildrenofisrael” was identified, but its incomplete subsequences, such as “hechildrenofisrael”, were not found in the results. Although the compound words “andthechildrenofisrael” and “ofthechildrenofisrael” also appeared in the results, their boundaries were also complete. Meanwhile, “thechildrenofisraela” in the results also indicated that this algorithm still needs to be improved.

**Table 3:** Top 20 longest words in the information spectrums of the *Holy Bible*

Words	Length	Words	Length
thetabernacleofthecongregation	30	thussaiththelordgod	19
andthechildrenofisrael	22	andthelordsaidunto	18
thelordspakeuntomoses	21	thehouseofthelord	17
ofthechildrenofisrael	21	rthussaiththelord	17
thechildrenofisraela	20	ethussaiththelord	17
anditshallcometopass	20	accordingtoallth	16
andthelordspakeuntom	20	eanditcametopass	16
saiththelordofhosts	19	thelordcommanded	16
anditcametopasswhen	19	ntothechildrenof	16
thechildrenofisrael	19	thehouseofisrael	16

### Algorithm verification with genomic comparison

**Fig. 2:**
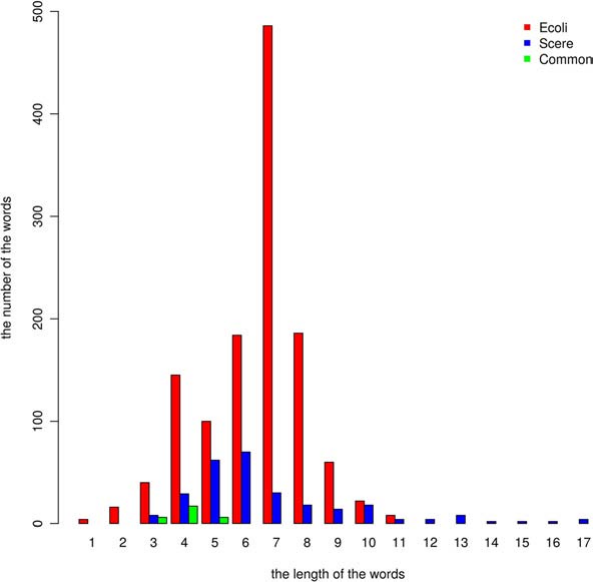
Comparison between Ecoli vocabulary and Scere vocabulary. The DNA words were sorted by length. The height of every bar represented the number of the words in a length. “Common” represented the common words between Ecoli and Scere

All DNA words were given in Additional file 4. As shown in Fig. 2, the length of most DNA words < 10. The word length ranged from 1 to 11 in Ecoli, and from 3 to 17 in Scere. In number, the words of Ecoli were larger than the words of Scere. Moreover, there were only a few common words between Ecoli and Scere. The difference showed in this figure demonstrated the great difference between the two species.

**Fig. 3:**
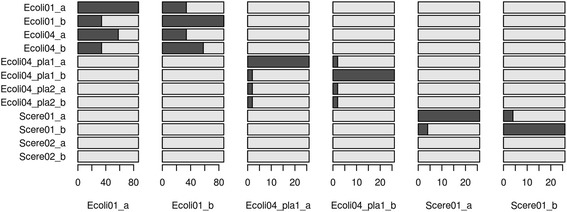
Comparison of vocabularies between the DNA strands. “pla” represented plasmid, “a” represented the sense strand, and “b” represented the antisense strand. Each column represented the data from a strand. The length of each bar represented the number of the words in a vocabulary and the length of the shaded part in each bar represented the words shared by two strands. Because the numbers of the words extracted from different strands were not the same, the horizontal scales were also different

Besides, the vocabularies of all DNA strands were compared. A complete comparison between each two strands was given in Additional file 5 (its legend was given in Additional file 6), and the partial results were shown in Fig. 3. As shown in Fig. 3, all genomes can be divided into three categories: Ecoli nuclear chromosomes, Ecoli plasmid chromosomes and Scere chromosomes. There were hardly common words among the three classes of chromosomes. We observed huge differences among them.

For the comparison among the chromosomes within each class, there was a high similarity among Ecoli nuclear chromosomes. There was a low similarity among plasmid chromosomes, and the similar results were showed for Scere chromosomes. For the comparison between the sense strand and the antisense strand of the same chromosome, there was a higher similarity between two strands of each Ecoli nuclear chromosomes. There was a low similarity between two strands of each plasmid chromosomes, and the similar results were shown for Scere chromosomes. As shown in these results, the vocabularies can show the features of different chromosome, and these DNA words had biological significances.

## Associations between DNA words and gene functions

**Fig. 4:**
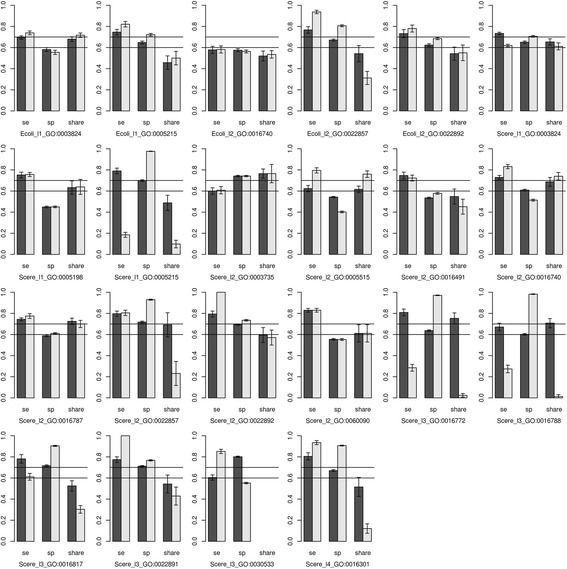
Evaluation of function prediction based on DNA words. “se”, “sp”, “share” indicated sensitivity, specificity, and the predicted accuracy rate in the share group, respectively. The black bar indicated the predicted effect without the interaction, and the white bar indicated the predicted effect with the first-order interaction

For each GO term, two fitting models were established depending on whether or not the first-order interaction was added. In the end, 22 GO terms were used to establish 44 fitting models. The predicted effects of all models were illustrated in Fig. 4, and detailed values were shown in Tables 4 and 5.

**Table 4:** Evaluation of function prediction of logistic model without interaction

Terms	Sensitivity	MAD	Specificity	MAD	Agreement rate	MAD	Share	MAD
Ecoli_l1_GO:0003824	0.695	0.016	0.581	0.018	0.638	0.014	0.680	0.022
Ecoli_l1_GO:0005215	0.746	0.026	0.648	0.013	0.657	0.011	0.457	0.064
Ecoli_l2_GO:0016740	0.578	0.034	0.575	0.014	0.577	0.014	0.520	0.047
Ecoli_l2_GO:0022857	0.767	0.031	0.671	0.010	0.681	0.009	0.542	0.077
Ecoli_l2_GO:0022892	0.732	0.038	0.623	0.013	0.633	0.011	0.542	0.062
Scere_l1_GO:0003824	0.734	0.011	0.651	0.011	0.683	0.007	0.653	0.030
Scere_l1_GO:0005198	0.752	0.027	0.449	0.008	0.471	0.006	0.633	0.063
Scere_l1_GO:0005215	0.790	0.027	0.699	0.008	0.706	0.008	0.488	0.072
Scere_l2_GO:0003735	0.600	0.031	0.742	0.007	0.737	0.007	0.765	0.044
Scere_l2_GO:0005515	0.623	0.030	0.543	0.006	0.550	0.008	0.616	0.031
Scere_l2_GO:0016491	0.747	0.032	0.535	0.007	0.545	0.008	0.548	0.071
Scere_l2_GO:0016740	0.729	0.019	0.609	0.008	0.622	0.007	0.687	0.042
Scere_l2_GO:0016787	0.744	0.014	0.588	0.009	0.608	0.009	0.725	0.030
Scere_l2_GO:0022857	0.797	0.025	0.718	0.009	0.722	0.007	0.692	0.114
Scere_l2_GO:0022892	0.794	0.028	0.694	0.006	0.699	0.006	0.595	0.071
Scere_l2_GO:0060090	0.830	0.018	0.554	0.007	0.575	0.008	0.611	0.082
Scere_l3_GO:0016772	0.809	0.032	0.637	0.007	0.646	0.007	0.753	0.052
Scere_l3_GO:0016788	0.671	0.036	0.602	0.008	0.604	0.009	0.708	0.044
Scere_l3_GO:0016817	0.781	0.040	0.716	0.008	0.719	0.008	0.525	0.049
Scere_l3_GO:0022891	0.774	0.027	0.712	0.006	0.713	0.008	0.543	0.085
Scere_l3_GO:0030533	0.604	0.025	0.801	0.006	0.788	0.005	NA	NA
Scere_l4_GO:0016301	0.805	0.034	0.670	0.009	0.678	0.005	0.515	0.090

**Table 5:** Evaluation of function prediction of logistic model with the first-order interaction

Terms	Sensitivity	MAD	Specificity	MAD	Agreement rate	MAD	Share	MAD
Ecoli_l1_GO:0003824	0.739	0.018	0.555	0.020	0.655	0.011	0.717	0.021
Ecoli_l1_GO:0005215	0.821	0.026	0.722	0.012	0.735	0.009	0.500	0.064
Ecoli_l2_GO:0016740	0.582	0.034	0.563	0.014	0.569	0.011	0.535	0.035
Ecoli_l2_GO:0022857	0.937	0.016	0.806	0.009	0.816	0.009	0.312	0.062
Ecoli_l2_GO:0022892	0.780	0.033	0.685	0.010	0.696	0.012	0.550	0.074
Scere_l1_GO:0003824	0.619	0.012	0.706	0.008	0.673	0.007	0.611	0.033
Scere_l1_GO:0005198	0.757	0.019	0.450	0.008	0.471	0.007	0.639	0.071
Scere_l1_GO:0005215	0.185	0.024	0.976	0.002	0.916	0.004	0.098	0.036
Scere_l2_GO:0003735	0.608	0.035	0.742	0.006	0.737	0.007	0.765	0.087
Scere_l2_GO:0005515	0.795	0.025	0.402	0.008	0.430	0.008	0.760	0.031
Scere_l2_GO:0016491	0.723	0.028	0.578	0.009	0.586	0.007	0.452	0.071
Scere_l2_GO:0016740	0.831	0.020	0.514	0.009	0.556	0.011	0.740	0.036
Scere_l2_GO:0016787	0.775	0.023	0.610	0.007	0.633	0.008	0.700	0.034
Scere_l2_GO:0022857	0.805	0.025	0.930	0.005	0.923	0.003	0.231	0.114
Scere_l2_GO:0022892	1.000	0.000	0.735	0.007	0.750	0.007	0.571	0.071
Scere_l2_GO:0060090	0.830	0.018	0.553	0.009	0.574	0.007	0.611	0.082
Scere_l3_GO:0016772	0.285	0.032	0.971	0.003	0.936	0.004	0.024	0.017
Scere_l3_GO:0016788	0.274	0.036	0.982	0.003	0.954	0.004	0.012	0.018
Scere_l3_GO:0016817	0.611	0.032	0.904	0.005	0.890	0.004	0.303	0.036
Scere_l3_GO:0022891	1.000	0.000	0.767	0.006	0.777	0.007	0.429	0.085
Scere_l3_GO:0030533	0.852	0.020	0.552	0.007	0.573	0.008	NA	NA
Scere_l4_GO:0016301	0.935	0.019	0.907	0.004	0.908	0.005	0.121	0.045

As shown in Fig. 4, without considering the interaction, the predicted sensitivity and specificity of the fitting models of Scere_l1_GO:0005215, Scere_l2_GO:0022857, Scere_l2_GO:0022892, Scere_l3_GO:0016817, and Scere_l3_GO:0022891 were greater than 0.7. With the first-order interaction, the predicted sensitivity and specificity of the fitting models of Ecoli_l1_GO:0005215, Ecoli_l2_GO:0022857, Scere_l2_GO:0022857, Scere_l2_GO:0022892, Scere_l3_GO:0022891, and Scere_l4_GO:0016301 were greater than 0.7. The functions of these GO terms are transporter activity (GO:0005215), transmembrane transporter activity (GO:0022857), substrate-specific transporter activity (GO:0022892), substrate-specific transmembrane transporter activity (GO:0022891), hydrolase activity on acid anhydrides (GO:0016817) and kinase activity (GO:0016301), respectively. These logistic models included the DNA words and corresponding coefficients related with the functions. Given a gene sequence, the frequency of all DNA words could be counted. Therefore, the probability prediction of these functions could be acquired according to corresponding models. It should be noted that a gene from prokaryote should be applied with the models of Ecoli, and a gene from eukaryote should be applied with the models of Scere.

Although the interaction can improve the prediction performance in many cases, but not always. In addition, the predicted results were bad in the share group. Although the predicted accuracy rates were high for some terms (e.g., Scere_l2_GO:0003735) in the share group, the sensitivity or specificity of these terms were poor and the prediction would be meaningless.

## Discussion

In this study, we hypothesize that non-uniform distribution and integrity were two important features of a DNA word. Therefore, we can define a word as a complete symbol sequence not following the uniform distribution within a certain scope. Carpena et al. [11] and Hackenberg et al. [12] have shown the importance of the non-uniform distribution in the identification of the words. Carpena et al. [11] have also explored the integrity with the help of the semantic meanings of the words, but their method can not be applied to the genome directly. In this study, we proposed a novel method with the help of the sequence and position alignment among the symbol sequences.

Negative control is very important for evaluating the false positive of an ab initio algorithm. In this study, two random base sequences were adopted to check it. As shown in the results, the denoising capability of our algorithm was reasonable. For the DNA vocabulary, there has not been a gold standard. Therefore, we used an English text, *Holy Bible*, as a positive control. As shown in the results, not only some words can be extracted, but also the boundary of many words can be identified. However, we also realized that the boundary of some words still cannot be identified accurately. Note that the symbol sequences were analyzed in a descending order for given word lengths in the algorithm. This unidirectional search can cause an amplification of biases. The results can be improved by a bidirectional search, which will be investigated in our future work. Moreover, the integrity was not quantitatively analyzed. We expect that a quantitative evaluation method independent of dictionary can be developed in the near future.

In this study, the great difference between two species, Ecoli and Scere, was illustrated by their vocabularies. Meanwhile, the similarities and differences between the DNA strands can also be clearly shown by their vocabularies. It should be noted that the antisense strand is the reverse complementary chain of the sense strand, but the information of two strands is different. From these comparison results, further utilization of this algorithm can be extended to the area of taxonomy.

In the results of the English text, it is obvious that not all English words were captured. In order to capture the missing DNA words in a genome study, a DNA vocabulary can be constructed by integrating all the DNA words extracted from different DNA strands. According to the DNA vocabulary and gene annotations, we explored the relationships between gene functions and the DNA words. The results showed that the words can predict gene functions to some degree. In many cases, prediction ability was improved with interaction, but not always. In this study, the product of two word frequencies was adopted in the inspection of the interaction. Other higher order interactions might exit and can be considered as well.

An important problem in the function prediction was that the prediction results were not satisfying in the share group. The reason might be that the functions of the share group were fundamentally different from the functions of exclusive group. In addition, there were many functions which were not able to be predicted by these words. It might indicate that these functions were not the major or critical features of the chromosomes.

## Conclusions

In summary, we proposed a novel definition for DNA word based on distribution and integrity. According to the definition, a simple and effective algorithm was developed to extract DNA words, based on which a DNA dictionary can be built ab initio. This may open a new perspective to explore the functions of a genome with the aid of computationally efficient tools.

## Additional files

Additional file 1:
**Link of Download.doc.** The download links of the data in this manuscript. (DOC 12 kb) Additional file 2:
**Numbers of Chromosomes.doc.** The numbers of all chromosomes were listed in this file. (DOC 45 kb) Additional file 3:
**Vocabulary of Holy Bible.csv.** The words extracted from the *Holy Bible* were listed in these files. (CSV 28 kb) Additional file 4:
**DNAWords.csv.** The DNA words extracted from Ecoli and Scere. (CSV 23 kb) Additional file 5:
**Complete Comparison of vocabularies.jpg.** A complete comparison between the vocabularies of all DNA strands was shown in this figure. (JPG 10477 kb) Additional file 6:
**The legend of Additional file** 5 **.doc.** (DOC 10 kb)

## Abbreviations

KS: Kolmogorov-Smirnov
Scere: Saccharomyces cerevisiae
Ecoli: Escherichia coli
