# Supplementary material for: Extracting DNA words based on the sequence features: non-uniform distribution and integrity
Source: Theor Biol Med Model. 2016 Jan 25;13:2. doi: 10.1186/s12976-016-0028-3 (PMC4727310; doi:10.1186/s12976-016-0028-3)
Supplement: Additional file 1: — Link of Download.doc. The download links of the data in this manuscript. (DOC 12 kb) [file 12976_2016_28_MOESM1_ESM.doc]

Link of Download

1 reference genome

1.1 Escherichia coli

ftp://ftp.ncbi.nih.gov/genomes/Bacteria/Escherichia_coli_K_12_substr__MG1655_uid57779/

ftp://ftp.ncbi.nih.gov/genomes/Bacteria/Escherichia_coli_K_12_substr__W3110_uid161931/

ftp://ftp.ncbi.nih.gov/genomes/Bacteria/Escherichia_coli_K_12_substr__DH10B_uid58979/

ftp://ftp.ncbi.nih.gov/genomes/Bacteria/Escherichia_coli_E24377A_uid58395/

ftp://ftp.ncbi.nih.gov/genomes/Bacteria/Escherichia_coli_O157_H7_EC4115_uid59091/

ftp://ftp.ncbi.nih.gov/genomes/Bacteria/Escherichia_coli_SE11_uid59425/

ftp://ftp.ncbi.nih.gov/genomes/Bacteria/Escherichia_coli_IAI1_uid59377/

ftp://ftp.ncbi.nih.gov/genomes/Bacteria/Escherichia_coli_B_REL606_uid58803/

ftp://ftp.ncbi.nih.gov/genomes/Bacteria/Escherichia_coli_BL21_DE3__uid161947/

ftp://ftp.ncbi.nih.gov/genomes/Bacteria/Escherichia_coli_KO11FL_uid52593/

1.2 Saccharomyces cerevisiae

ftp://ftp.ncbi.nih.gov/genomes/Fungi/Saccharomyces_cerevisiae_uid128/

2 gene annotation

2.1 Ontology file

http://www.geneontology.org/ontology/obo_format_1_2/gene_ontology_ext.obo

2.2 annotation file

http://viewvc.geneontology.org/viewvc/GO-SVN/trunk/gene-associations/gene_association.ecocyc.gz?rev=HEAD

http://viewvc.geneontology.org/viewvc/GO-SVN/trunk/gene-associations/gene_association.sgd.gz?rev=HEAD
