# Supplementary material for: Extracting DNA words based on the sequence features: non-uniform distribution and integrity
Source: Theor Biol Med Model. 2016 Jan 25;13:2. doi: 10.1186/s12976-016-0028-3 (PMC4727310; doi:10.1186/s12976-016-0028-3)
Supplement: Additional file 2: — Numbers of Chromosomes.doc. The numbers of all chromosomes were listed in this file. (DOC 45 kb) [file 12976_2016_28_MOESM2_ESM.doc]

Numbers of Chromosomes

| No. | ID | Chromosomes |
| --- | --- | --- |
| Ecoli01.fna | NC_000913.2 | Escherichia coli str. K-12 substr. MG1655 chromosome |
| Ecoli02.fna | NC_007779.1 | Escherichia coli str. K-12 substr. W3110 |
| Ecoli03.fna | NC_010473.1 | Escherichia coli str. K-12 substr. DH10B chromosome |
| Ecoli04.fna | NC_009801.1 | Escherichia coli E24377A chromosome |
| Ecoli04_pla1.fna | NC_009786.1 | Escherichia coli E24377A plasmid pETEC_80e |
| Ecoli04_pla2.fna | NC_009787.1 | Escherichia coli E24377A plasmid pETEC_35e |
| Ecoli04_pla3.fna | NC_009788.1 | Escherichia coli E24377A plasmid pETEC_73e |
| Ecoli04_pla4.fna | NC_009789.1 | Escherichia coli E24377A plasmid pETEC_6e |
| Ecoli04_pla5.fna | NC_009790.1 | Escherichia coli E24377A plasmid pETEC_74e |
| Ecoli04_pla6.fna | NC_009791.1 | Escherichia coli E24377A plasmid pETEC_5e |
| Ecoli05.fna | NC_011353.1 | Escherichia coli O157:H7 str. EC4115 chromosome |
| Ecoli05_pla1.fna | NC_011350.1 | Escherichia coli O157:H7 str. EC4115 plasmid pO157e |
| Ecoli05_pla2.fna | NC_011351.1 | Escherichia coli O157:H7 str. EC4115 plasmid pEC4115e |
| Ecoli06.fna | NC_011415.1 | Escherichia coli SE11 chromosome |
| Ecoli06_pla1.fna | NC_011407.1 | Escherichia coli SE11 plasmid pSE11-4e |
| Ecoli06_pla2.fna | NC_011408.1 | Escherichia coli SE11 plasmid pSE11-5e |
| Ecoli06_pla3.fna | NC_011411.1 | Escherichia coli SE11 plasmid pSE11-6e |
| Ecoli06_pla4.fna | NC_011413.1 | Escherichia coli SE11 plasmid pSE11-2e |
| Ecoli06_pla5.fna | NC_011416.1 | Escherichia coli SE11 plasmid pSE11-3e |
| Ecoli06_pla6.fna | NC_011419.1 | Escherichia coli SE11 plasmid pSE11-1e |
| Ecoli07.fna | NC_011741.1 | Escherichia coli IAI1 chromosome |
| Ecoli08.fna | NC_012967.1 | Escherichia coli B str. REL606 chromosome |
| Ecoli09.fna | NC_012971.2 | Escherichia coli BL21(DE3) chromosome |
| Ecoli10.fna | NC_016902.1 | Escherichia coli KO11FL chromosome |
| Ecoli10_pla1.fna | NC_016903.1 | Escherichia coli KO11FL plasmid pEKO1102e |
| Ecoli10_pla2.fna | NC_016904.1 | Escherichia coli KO11FL plasmid pEKO1101e |
| Scere01.fna | NC_001133.9 | Saccharomyces cerevisiae S288c chromosome Ie |
| Scere02.fna | NC_001134.8 | Saccharomyces cerevisiae S288c chromosome IIe |
| Scere03.fna | NC_001135.5 | Saccharomyces cerevisiae S288c chromosome IIIe |
| Scere04.fna | NC_001136.10 | Saccharomyces cerevisiae S288c chromosome IVe |
| Scere05.fna | NC_001137.3 | Saccharomyces cerevisiae S288c chromosome Ve |
| Scere06.fna | NC_001138.5 | Saccharomyces cerevisiae S288c chromosome VIe |
| Scere07.fna | NC_001139.9 | Saccharomyces cerevisiae S288c chromosome VIIe |
| Scere08.fna | NC_001140.6 | Saccharomyces cerevisiae S288c chromosome VIIIe |
| Scere09.fna | NC_001141.2 | Saccharomyces cerevisiae S288c chromosome IXe |
| Scere10.fna | NC_001142.9 | Saccharomyces cerevisiae S288c chromosome Xe |
| Scere11.fna | NC_001143.9 | Saccharomyces cerevisiae S288c chromosome XIe |
| Scere12.fna | NC_001144.5 | Saccharomyces cerevisiae S288c chromosome XIIe |
| Scere13.fna | NC_001145.3 | Saccharomyces cerevisiae S288c chromosome XIIIe |
| Scere14.fna | NC_001146.8 | Saccharomyces cerevisiae S288c chromosome XIVe |
| Scere15.fna | NC_001147.6 | Saccharomyces cerevisiae S288c chromosome XVe |
| Scere16.fna | NC_001148.4 | Saccharomyces cerevisiae S288c chromosome XVIe |
| Scere_mit.fna | NC_001224.1 | Saccharomyces cerevisiae S288c mitochondrion |
