# Supplementary material for: Extracting DNA words based on the sequence features: non-uniform distribution and integrity
Source: Theor Biol Med Model. 2016 Jan 25;13:2. doi: 10.1186/s12976-016-0028-3 (PMC4727310; doi:10.1186/s12976-016-0028-3)
Supplement: Additional file 6: — The legend of Additional file 5 .doc. (DOC 10 kb) [file 12976_2016_28_MOESM6_ESM.doc]

"pla" represented plasmid, "a" represented the sense strand, and "b" represented the antisense strand. Each column represented the data from a strand. The length of each bar represented the number of the words in the vocabulary of a strand and the length of the shaded part in each bar represented the words shared by two strands. Because the numbers of the words extracted from different strands were not the same, the horizontal scales were also different.
